# Plasmonically induced perfect absorption in graphene/metal system

**DOI:** 10.1186/s11671-019-3121-9

**Published:** 2019-08-28

**Authors:** Cheng Hu, Qi Lin, Xiang Zhai, Mengting Wen, Lingling Wang

**Affiliations:** Key Laboratory for Micro/Nano Optoelectronic Devices of Ministry of Education and Hunan Provincial Key Laboratory of Low-Dimensional Structural Physics and Devices, School of Physics and Electronics, Hunan University, Changsha, 410082 China

**Keywords:** Plasmonically induced absorption, Graphene, Perfect absorption

## Abstract

The constructive interference of bright and dark plasmonic modes results in plasmon-induced absorption (PIA) effect. Here, we theoretically investigate PIA effect, which is realized by the constructive interference between a Fabry-Perot (F-P) resonance mode and a graphene quasi-guided mode. Numerical simulation reveals at least three advantages of our structure over previous ones. First, the extinction ratio can reach ~ 99.999%, resulting in the ultra-high figure of merit* (FOM*) as high as 10^6^. Second, the intensity of this pronounced PIA effect can be optimized by adjusting the coupling distance. Third, the resonance frequency can be easily tunable by tuning the graphene Fermi level. This system may have potential applications in dynamically optical switching and biochemical sensing.

**Electronic supplementary material:**

The online version of this article (10.1186/s11671-019-3121-9) contains supplementary material, which is available to authorized users.

## Background

Plasmonics has attracted wide attention due to its extraordinary properties [1–15] and huge potential in many fields, including integrated photonics, bio-sensing, energy capture, photodetection. Recently, a novel plasmonic phenomenon, known as the spoof surface plasmons (SSPs), has been observed, which can propagate through perforated metals and overcome the diffraction limit [16]. SSPs were then explored in the THz, microwave, and lower frequency range [17–19], and a number of deep-subwavelength devices based on SSPs have been proposed demonstrated [20, 21]. However, the application of such devices is seriously hampered by SPPs’ high damping rate. One solution to this issue is the artificial plasmon-induced transparency (PIT) medium [22], which features a sharp transparency window within a broad absorption spectrum. The PIT effect mainly relies on the coupling of a radiative element and a subradiant element, which has been widely studied [23–25]. A similar phenomenon, plasmon-induced absorption (PIA), has also been demonstrated recently, which results from the constructive interference of bright and dark plasmonic modes [26]. The PIA resonance [27, 28] can exhibit remarkably fast-light effect, which has potential applications in optical switching and processing.

However, traditional devices based on the PIA effect of the metallic structure are hard or impossible to obtain tunability, which seriously restricts its applications. Graphene [29, 30], known for its semi-metallicity, high mobility, and high tunability, can be an excellent candidate material for tunable infrared plasmonic devices. In this paper, we investigated a tunable PIA effect, which is achieved by the constructive interference of an F-P resonance mode and a quasi-guided mode supported by a periodic silver groove and monolayer graphene respectively. It is found that the resonance strength and linewidth are strongly dependent on coupling distance. It is also shown that the extinction ratio can reach ~ 99.999%. The extinction ratio is defined as 1-*R*-*T*, where *R* and *T* are the reflectance and transmittance, respectively. It is simply 1-*R* in our system since the transmittance here is 0. As a result, an ultra-high FOM* as high as 10^6^ in the graphene/metal system can be achieved and the resonance frequency can be dynamically tunable by adjusting the gate voltage of graphene. These prominent properties can be applied in biochemical sensing and dynamically optical switching.

## Methods

**Fig. 1:**
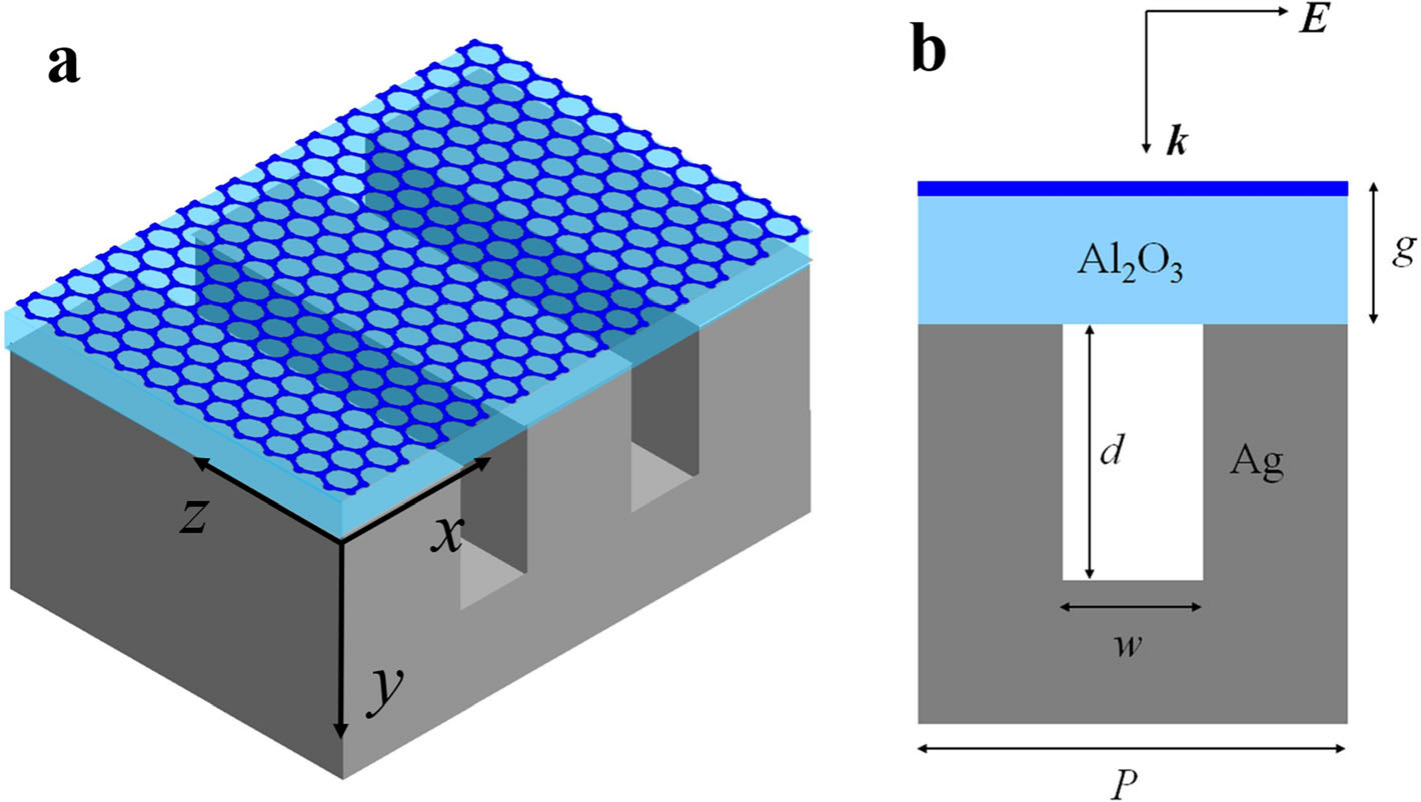
Schematic diagram of the graphene-silver groove structure. **a** Oblique view. **b** The cross-section diagram of a unit cell

The schematic of our structure is shown in Fig. 1, consisting of a monolayer graphene and an Al_2_O_3_ isolated layer on top of a grooved silver. The thickness of the Al_2_O_3_ is *g*. The system is illuminated by a normal-incident plane wave of transverse magnetic (TM) polarization. The other structural parameters are expressed as follows: *d* is the depth of the silver groove; *w* is the width of the silver groove; *P* is the period of the unit cell. In the mid-infrared region, intraband scattering dominates in highly doped graphene, and its conductivity takes on a Drude-like form *σ*_*g*_ = *ie*^2^*E*_F_/[*πħ*^2^(*ω*+*iτ*^-1^)]. The electron relaxation time is expressed as *τ* = *μE*_F_/*eυ*_F_^2^, where *υ*_F_ = *c*/300 is the Fermi velocity, *E*_F_ is the Fermi energy and *μ* = 10 m^2^/Vs is the DC mobility of graphene [25, 31, 32]. In the finite-difference time-domain (FDTD) simulations, the optical constants for silver, and Al_2_O_3_ are from ref. [33] and ref. [34]. The periodic boundary conditions are used to simulate infinite periodic cell structures. For simplicity, we assume the material of the region above the graphene layer is vacuum (*ε*_0_ = 1).

## Results and Discussion

**Fig. 2:**
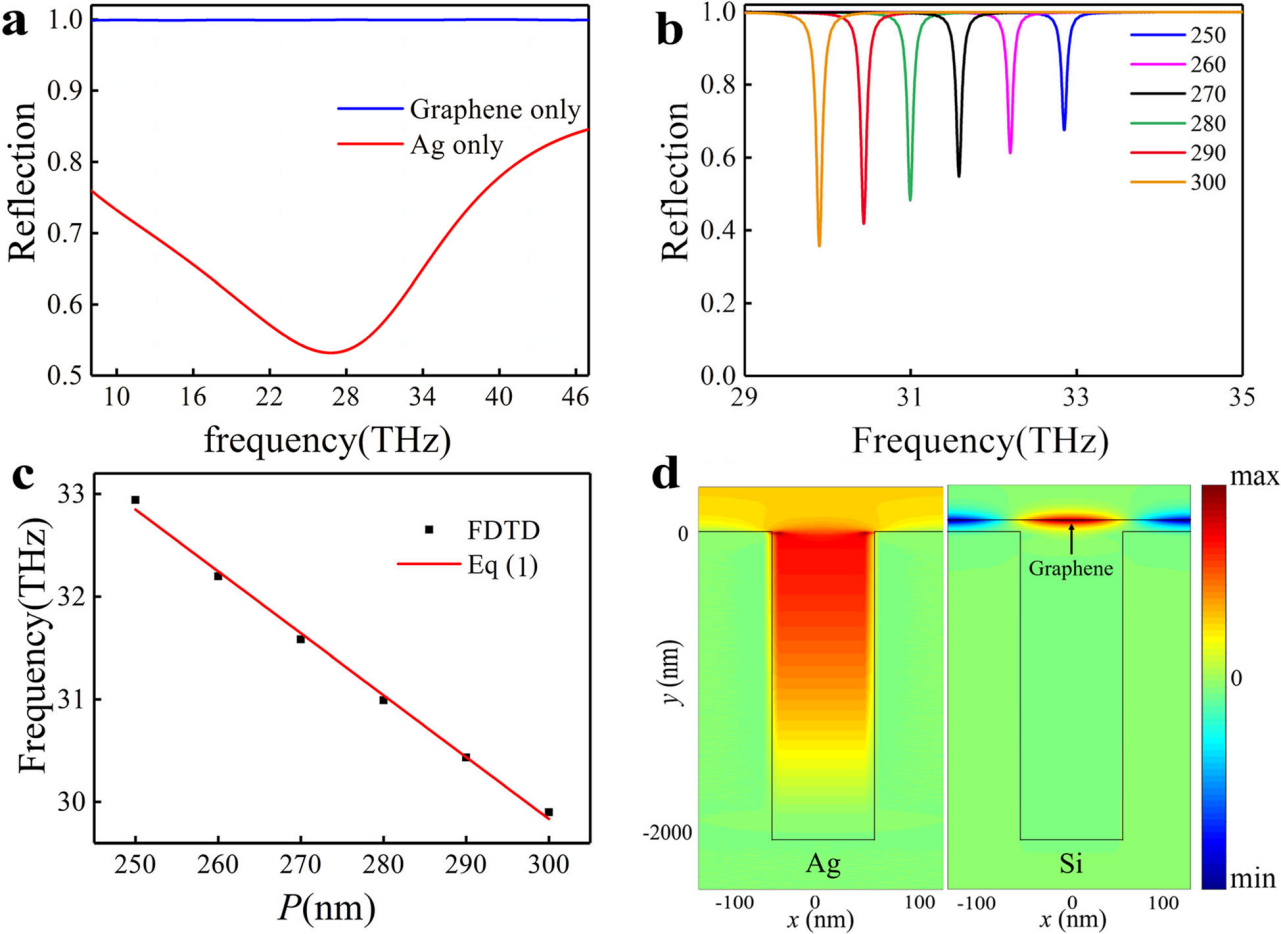
Optical response of the single modes. **a** The reflectance spectra of the structure of silver groove only (red line) and graphene only (blue line) in normal incidence, respectively. **b** The reflectance spectra of the structure of graphene-Si groove for different period *P* of unit cell. **c** The numerical modeling and analytical results of the resonant frequency *f*, respectively. **d** The electric field *E*_x_ distributions of F-P mode (left) and graphene quasi-guided resonance mode (right).

We simulated the reflection spectrum of the silver groove with *w* = 100 nm, *P* = 250 nm, *d* = 2000 nm, and the result is shown in Fig. 2a (red curve). A wide dip can be observed at ~ 28 THz, with an extinction ratio ~ 44% and *Q* factor ~ 0.8, which is due to an F-P resonance induced by the SSP excited by incident light [19]. This resonance has a wide range of resonance bands and thus the resonance mode can serve as the superradiant mode in our PIA system. Then, we calculated the reflection spectrum of the graphene sheet with metal boundary conditions in the bottom of the simulation area, with a Fermi level *E*_F_ = 0.3 eV, as shown in Fig. 2a (blue curve). The reflection spectrum shows that the graphene plasmon resonance can not be directly excited by the incidence at this frequency. To visualize and optimize the plasmon mode supported by the graphene, we first simulate the resonance modes supported by the graphene. To eliminate the potential impact of the silver groove’s F-P resonance, we assume the groove is made of silicon instead of silver. The reflectance spectra of the structure were calculated for *E*_F_ = 0.3 eV and different unit cell *P* and is shown in Fig. 2b. A reflectance dip at resonant frequency *f* = 32.84 THz can be observed for *P* = 250 nm with a *Q* factor ~ 304. The high *Q* resonance with a narrow resonance band can serve as the subradiant (dark) mode in our PIA system. The reflectance dip is due to the resonance of plasmonic quasi-guided mode in graphene with the normal incidence [35] since the groove can compensate the wavevector mismatch based on the *m*th order phase-matching condition [36, 37]

1 $$ {k}_0\operatorname{Re}\left({n}_{\mathrm{eff}}\right)=\left|{k}_x+{mG}_x\right|,, $$

where *k*_*x*_ = *k*_0_sin*θ*, *k*_0_ = 2*π*/*λ* is the wavevector in free space, *θ* is the angle between the incident light and *y*-direction, *n*_eff_ is the effective refractive index of TM waveguide mode in the graphene, and *G*_x_ is the reciprocal lattice vector of the grating (*G*_x_ = 2*π*/*P*). In the following discussion, the incident light angle of *y*-direction is zero (*θ* = 0°). The situation for other incident angles is discussed in Additional file 1. The positions of these reflectance dips correspond to the resonance frequency of the quasi-guided mode in the graphene, as shown in Fig. 2b. The simulation results agree well with the Eq. (1), where *m* = 1 and the effective refractive index, ~ 33, is obtained by FDTD solutions, as shown in Fig. 2c. The electric field *E*_x_ distributions of F-P mode and graphene quasi-guided mode have been shown in Fig. 2d. It is noted that the energy confinement of the SSP modes sustained by the Si grooved surface can be negligible compared with the graphene quasi-guided mode.

**Fig. 3:**
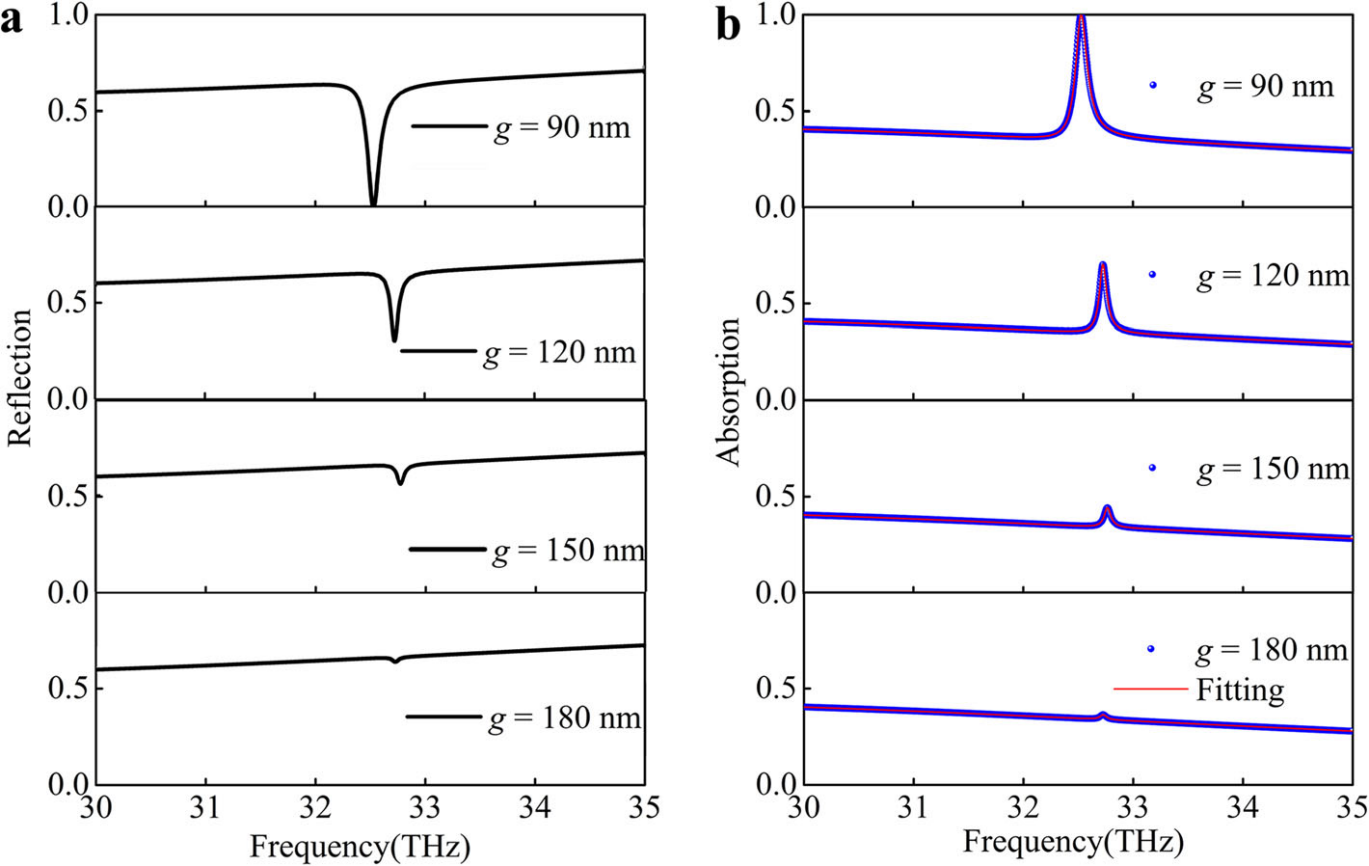
The optical response varies with the coupling distance. **a** The reflection. **b** Absorption spectra of the structure of graphene-silver groove in normal incidence for different distance *g* between the graphene and the silver groove. The black curve/the blue balls are calculated by FDTD method and the red curve is analytical fit by Eq. (3) of the PIA device

In the coupling situation, the two eigenmodes will be strongly coupled when they get close to each other, and hence the reflection spectrum will be dramatically changed. A narrow, sub-linewidth dip of enhanced absorbance with an extinction ratio ~ 99.97% is observed on top of the broader reflectance dip, as shown in Fig. 3a. When increasing the vertical distance *g*, the near-field coupling and the quasi-guided mode become weaker, as the modulation of the reflectance dip becomes smaller. There are two possible ways caused the reflectance dip smaller what are the weaker coupling and the weaker quasi-guided mode excitation. Therefore, we used the coupled oscillator model to quantitatively understand the PIA system [38].

2 $$ \left(\begin{array}{c}{\tilde{a}}_1\\ {}{\tilde{a}}_2\end{array}\right)=-{\left(\begin{array}{cc}\left(\omega -{\omega}_1+\frac{i{\gamma}_1}{2}\right)& \tilde{\kappa}\\ {}\tilde{\kappa}& \left(\omega -{\omega}_2+\frac{i{\gamma}_2}{2}\right)\end{array}\right)}^{-1}\left(\begin{array}{c}b{\tilde{E}}_0\\ {}0\end{array}\right) $$

Where ${\tilde{a}}_{1,2}={a}_{1,2}\left(\omega \right){\mathrm{e}}^{i\omega t}$, *ω*_1,2_ and *γ*_1,2_ are the time-harmonic amplitudes, resonant frequencies, and damping constants of the bright mode and the dark mode, respectively. *b* is the coupling coefficient measuring how strong the bright mode couples with the incident electric field. $\tilde{\kappa}=\kappa {e}^{i\varphi}$ is a complex coupling parameter, which is introduced to express the phase retardation effect. *φ* is a phase shift, which is a key coefficient to determine the form of the interference between the two coherent pathways. When *φ* = 0 is a real parameter and the typical behavior of the PIT effect can be observed, and the interference between the two coherent pathways is destructive. For *φ* = π/2 is a pure imaginary parameter and the interference between the two coherent pathways is converted from destructive to constructive [26]. The absorption of the system can be calculated as the dissipated energy on the basis of formula (2), which is

3 $$ A\left(\omega \right)=\Im \left(\frac{b\left(\omega -{\omega}_2+\frac{i{\gamma}_2}{2}\right)}{\kappa^2{e}^{i2\varphi }-\left(\omega -{\omega}_1+\frac{i{\gamma}_1}{2}\right)\left(\omega -{\omega}_2+\frac{i{\gamma}_2}{2}\right)}\right) $$

**Fig. 4:**
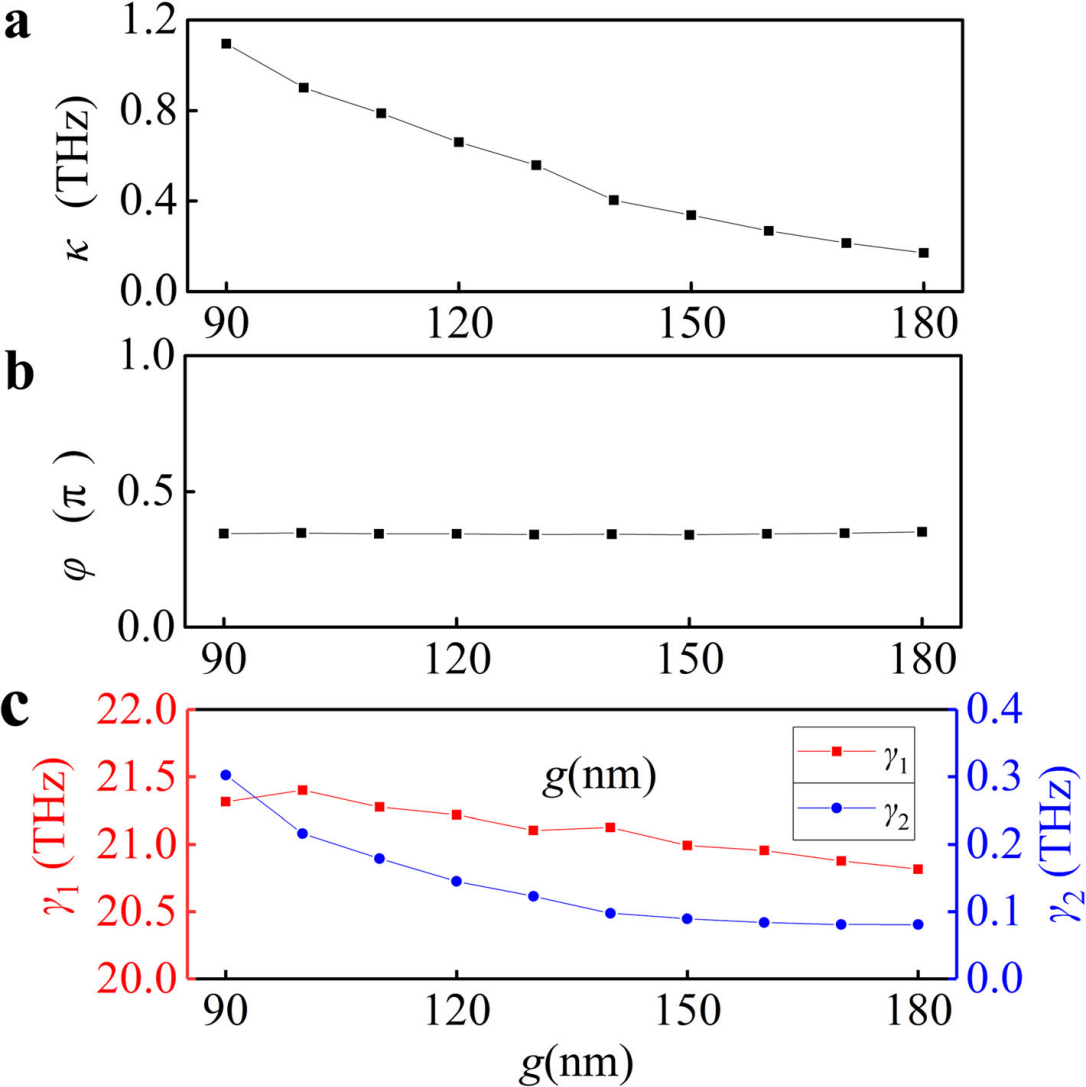
Quantitative analysis of optical responses in coupled systems. Extracted numerical (**a**) coupling, (**b**) phase, and (**c**) damping coefficients as a function of gap *g*. Values of *κ*, *φ*, and *γ*_1_, *γ*_2_ were extracted by fitting the numerical absorption spectra

Then, we fit the numerical absorption spectra with the Eq. (3) for different *g*, which have been shown in Fig. 3b (red curves). The simulation results are in good agreement with the analytical modeling results based on the coupled oscillator model, which strongly confirms the design principle of our PIA device. The fitting parameters *κ*, *φ*, *γ*_1_, and *γ*_2_ have been shown in Fig. 4a–c. The increasing *g* yields a decrease in the coupling parameter *κ*, as shown in Fig. 4a. When gradually decreasing the coupling (increasing *g*), the phase *φ* is unchanged, and *γ*_2_ gradually decreases while *γ*_1_ changes slightly shown in Fig. 4b, c. The coupling parameter *κ* exceed the damping constants of dark mode *γ*_2_ for the minimum gap distance, which confirms that the coupling from the bright mode to the dark mode is stronger than the dissipation processes in the graphene sheet.

**Fig. 5:**
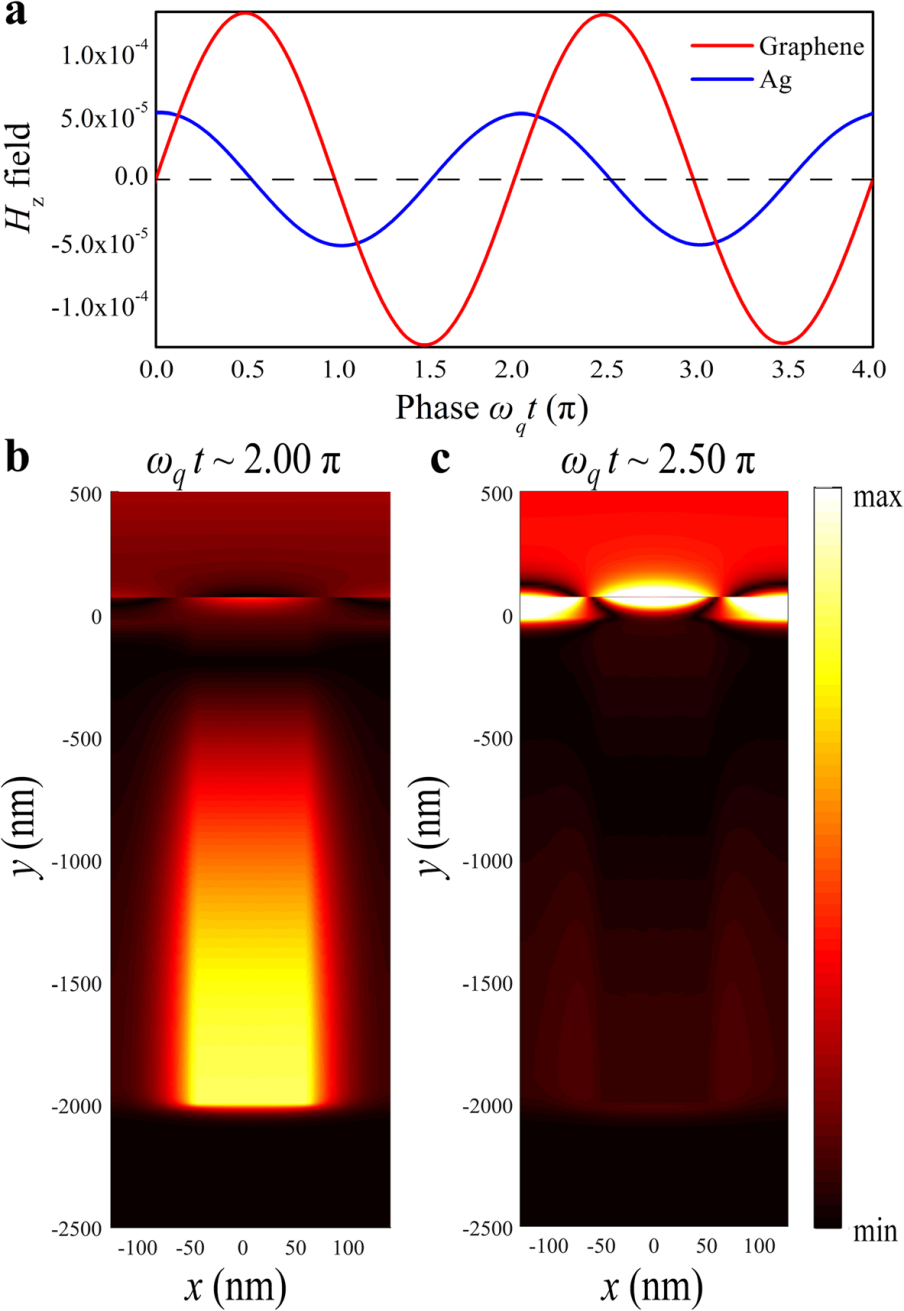
Time-domain evolution of coupled modes. **a** Calculated time evolution of the magnetic field strength at the graphene (red line) and silver groove (blue line). Calculated *z* component of the magnetic field distributions for *g* = 90 nm. Maximum field strength at silver groove and graphene are observed at different time **b** *ω*_q_*t*_1_ ~ 2.00π and **c** *ω*_q_*t*_2_ ~ 2.50π, respectively

To visualize the constructive interference between the bright and dark modes, we investigated the structure’s magnetic field evolution with the time, and two *H*_z_ monitors have been placed 3 nm away from the center of graphene and 1000 nm away from the bottom of the silver groove, respectively. The oscillating phase difference between the two modes is 0.5π, as indicated in Fig. 5a. The magnetic field distribution at a different time was calculated in the PIA resonance frequency *f*_q_ = 32.5 THz, where *ω*_q_*t*_1_ ~ 2.00 π and *ω*_q_*t*_2_ ~ 2.50π, as indicated in Fig. 5b, c. The maximum of the magnetic field in the silver groove can be observed for 2.00π while the magnetic field in graphene reaches its maximum for 2.50π, indicating the out-of-phase coupling between the two structures. Therefore, the evolution and formation of the resonance are determined by constructive interference [39].

**Fig. 6:**
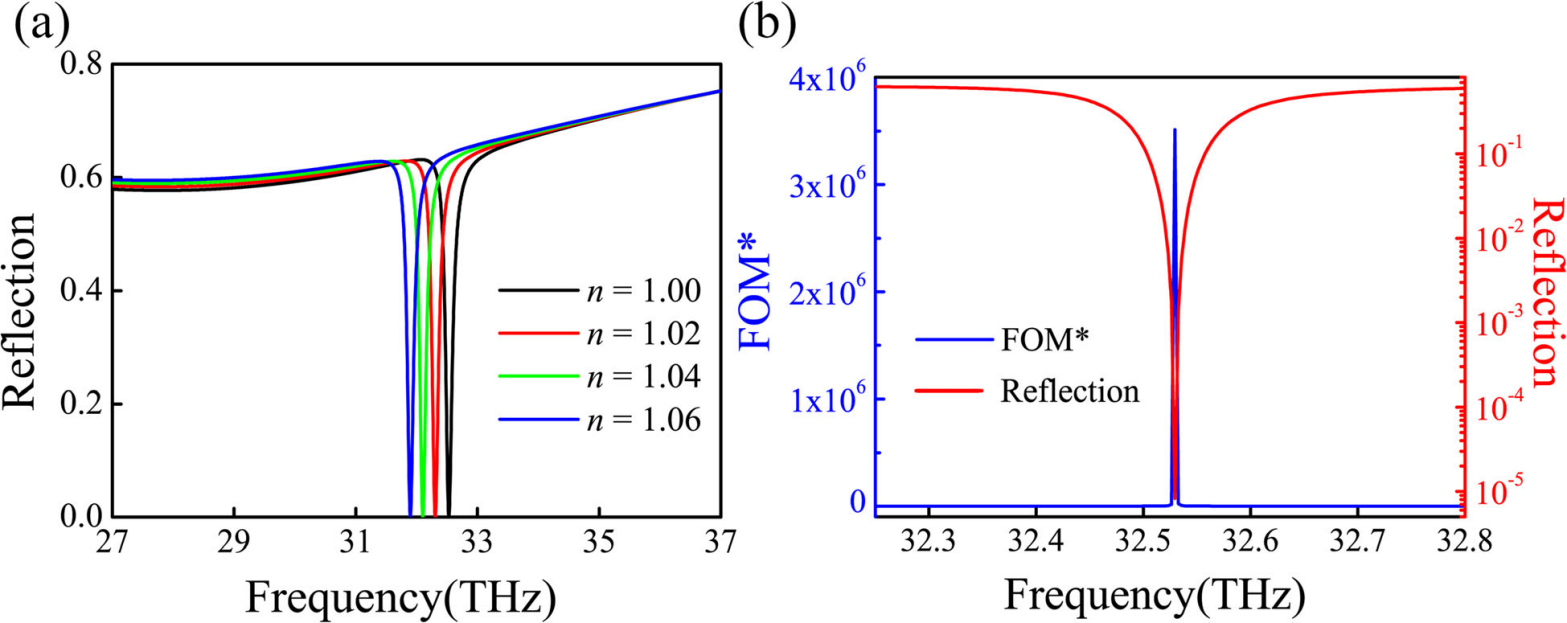
The sensing performance of the system. **a** The sensing response of the PIA sensor for varied dielectric environment. **b** The related FOM* curve and the reflectance spectra

In practical application, a narrow reflection band and high extinction ratio are highly desired. To achieve these two conditions, we can adjust period of unit *P* and depth of silver groove *d* to optimize our structural parameters. After calculating the reflection spectrum of different structure parameters *P* from 1900 to 2100 nm and *d* from 245 to 265 nm by FDTD, we earn a very high extinction ratio ~ 99.999% in *P* = 254 nm and *d* = 1980 nm. The reflectance spectrum of the PIA device under different refractive index environments is shown in Fig. 6a. The sensing capabilities are defined as [39]:

4 $$ {\displaystyle \begin{array}{c}S=\Delta f(THz)/\Delta n, FOM=S/ FWHM\ (THz),\\ {}S\ast =\Delta I/\Delta n, FOM\ast =S\ast /I,\end{array}} $$

where *f* and *I* are the resonance frequency and the spectral intensity, respectively. When measuring the reflection intensity of a sensor, the sensitivity capability of the sensor can be quantified by the FOM* value. The higher the value means the higher the sensitivity of the sensor. From Fig. 6 a, we can get *S* = 11.2 THz/RIU and the associated FOM~94.1, with the full width at half maximum (FWHM) ~ 30 nm (0.12 THz). This FOM is larger than the value in metamaterial absorbers based on surface lattice resonance. Also, our PIA sensor can lead to an ultra-high FOM* value 3.5 × 10^6^, as indicated in Fig. 6b. We compared the performance of the recently studied sensors in Additional file 1: Table S1.

In the PIA system, graphene plays another key role. The modulation of the resonant frequency can be achieved by tuning the gating voltage to adjust the Fermi level of graphene. The simulated spectra are shown in Additional files 2: Figure S1 and 3: Figure S2. The frequency-shift active control of the PIA resonance is meaningful for sensor or absorber.

## Conclusions

In summary, we have numerically demonstrated the perfect absorption induced by constructive interference between F-P resonance mode and graphene plasmonic quasi-guided mode. Through the introduction of graphene plasmonic quasi-guided mode, we obtain the spectral line with a narrower linewidth of the silver groove F-P resonance mode. When the distance *g* is gradually increased, the resonance strength and linewidth will decrease. For the application, the FOM* in our system can achieve 10^6^. Furthermore, the absorption window can be tuned by varying the geometrical parameter and the graphene Fermi level. These results could provide a new way toward the realization of nanoscale mid-infrared dynamical spectral control and ultrasensitive optical sensors.

## Additional files

Additional file 1: **Table S1.** The compare of the recent relevant works. (DOCX 19 kb)

Additional file 2: **Figure S1.** The reflectance spectra of the graphene-silver groove structure at different incident angle *θ. (PNG 236 kb)*

Additional file 3: **Figure S2.**The reflectance spectra of the structure of graphene-silver groove in normal incidence for different Fermi energy of the graphene sheet. (PNG 523 kb)

## Data Availability

All data generated or analyzed during this study are included in this published article [and its supplementary information files].

## Abbreviations

FDTD: Finite-difference time-domain
FOM*: Figure of merit*
F-P: Fabry-Perot
FWHM: Full width at half maximum
PIA: Plasmon-induced absorption
PIT: Plasmon-induced transparency
*Q* factor: Quality factor
SSPs: Spoof surface plasmons
TM: Transverse magnetic
