# Supplementary material for: Plasmonically induced perfect absorption in graphene/metal system
Source: Nanoscale Res Lett. 2019 Aug 28;14:300. doi: 10.1186/s11671-019-3121-9 (PMC6713770; doi:10.1186/s11671-019-3121-9)
Supplement: Supplementary file 1 — Table S1. The compare of the recent relevant works. (DOCX 19 kb) [file 11671_2019_3121_MOESM1_ESM.docx]

Supplementary Materials for

**Perfect absorption induced by** **plasmonically induced absorption in graphene/metal system**

Cheng Hu, Qi Lin, Xiang Zhai*, Mengting Wen, Lingling Wang

1. Different incident angles in the graphene/metal system

Now, we discuss the situation with different incident angle *θ* in the graphene/metal system. According to eq (1), we can obtain two solutions of *k*_0_ satisfying the phase-matching condition, as *θ*≠0°. As a result, two modes can be supported by the graphene sheet in the same *m* order. It can be verified by calculating the reflectance spectra of the system with different incident angle *θ*, as shown in Fig. S1. The new tiny reflection valley appears nearby the previous reflection valley, when *θ* = 5°. The difference between two reflection valleys gradually increases as increasing the incidence angle.

2. Tunable resonance frequency in our system

We demonstrate that plasmonically induced absorption (PIA) can be achieved by using a graphene/metal system. Frequency-shift active control of the PIA resonance is realized by varying the Fermi energy of the graphene without reoptimizing and refabricating the nanostructures. The reflectance spectra of the system in normal incidence for different Fermi energy of the graphene sheet has been shown in Fig. S2.

3. Compare with the recent research.

Table. S1. The compare of the recent relevant works.

| Year | FOM | FOM* | Tunability | Sensitivity (*S*) |
| --- | --- | --- | --- | --- |
| 2010 [39] | - | 87 | × | ~40 THz/RIU |
| 2015 [34] | 172 | 10^5^ | × | ~180 THz/RIU |
| 2016 [36] | 271 | - | √ | ~20 THz/RIU |
| 2017 [2] | 56.5 | - | × | ~130 THz/RIU |
| 2017 [S1] | 233.5 | 3×10^5^ | × | ~225 THz/RIU |
| 2018 [S2] | 12 | - | √ | ~4 THz/RIU |
| 2018 [S3] | ~10 | - | √ | ~0.1THz/RIU |
| This work | 94.1 | 3.5×10^6^ | √ | 11.2 THz/RIU |

**Reference**

[S1] D.Wu *et al*., “Ultra-narrow Band Perfect Absorber and Its Application as Plasmonic Sensor in the Visible Region,” *Nanoscale Research Letters,* vol. 12, Jun 26, 2017.

[S2] Y. Li et al., “Active control of an edge-mode-based plasmon-induced absorption sensor,” Applied Optics, vol. 57, no. 10, pp. 2698-2703, Apr 1, 2018.

[S3] Y. Xiang et al., “Tunable Dual-Band Perfect Absorber Based on L-Shaped Graphene Resonator,” Ieee Photonics Technology Letters, vol. 31, no. 6, pp. 483-486, Mar 15, 2019.
