# Supplementary figures and images for: Plasmonically induced perfect absorption in graphene/metal system
Source: Nanoscale Res Lett. 2019 Aug 28;14:300. doi: 10.1186/s11671-019-3121-9 (PMC6713770; doi:10.1186/s11671-019-3121-9)

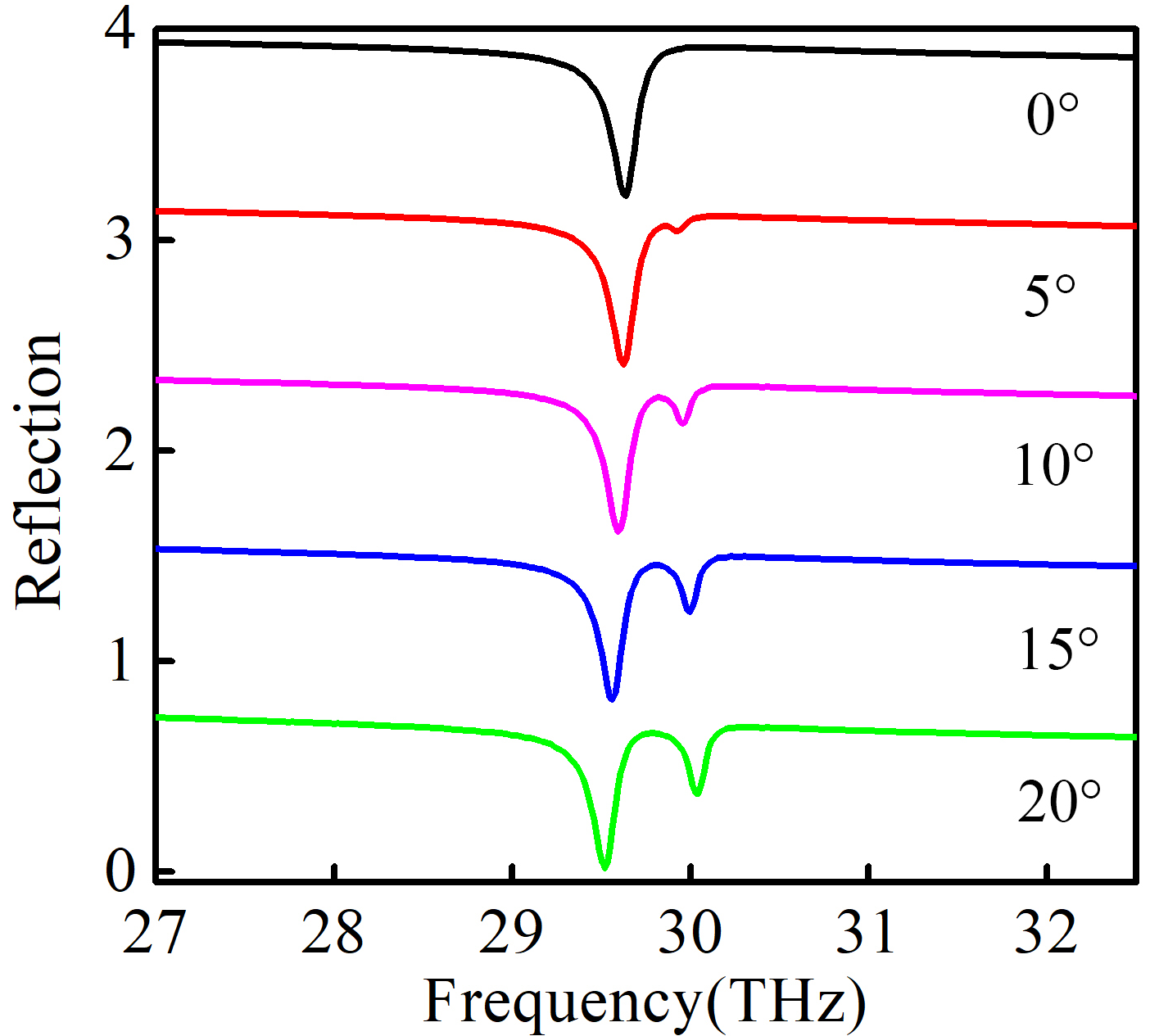

Supplement: Supplementary file 2 — Figure S1. The reflectance spectra of the graphene-silver groove structure at different incident angle θ. (PNG 236 kb) [file 11671_2019_3121_MOESM2_ESM.png]

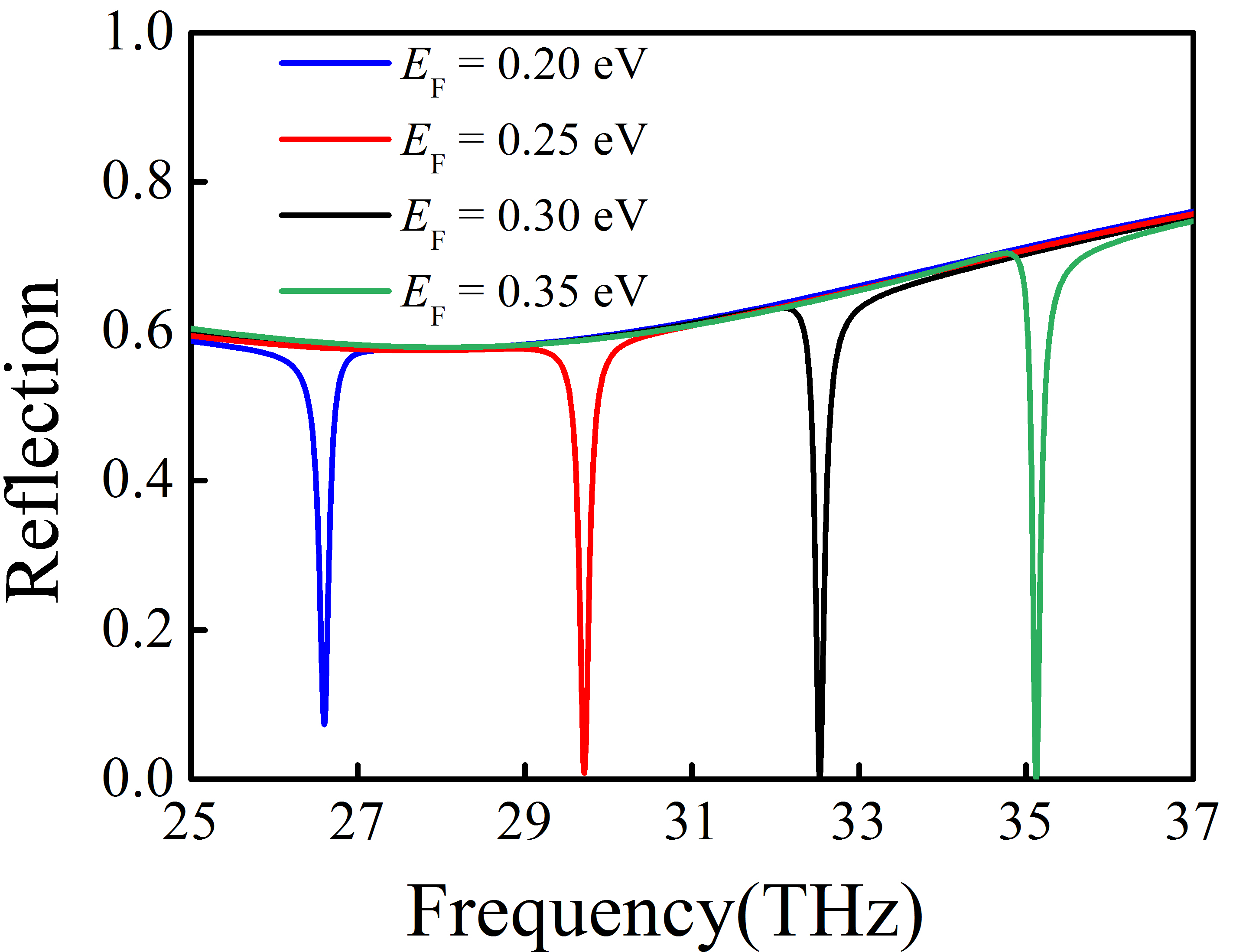

Supplement: Supplementary file 3 — Figure S2.The reflectance spectra of the structure of graphene-silver groove in normal incidence for different Fermi energy of the graphene sheet. (PNG 523 kb) [file 11671_2019_3121_MOESM3_ESM.png]
